# Targeting epigenetic modulation of cholesterol synthesis as a therapeutic strategy for head and neck squamous cell carcinoma

**DOI:** 10.1038/s41419-021-03760-2

**Published:** 2021-05-13

**Authors:** Xing Xu, Jun Chen, Yan Li, Xiaojie Yang, Qing Wang, Yanjun Wen, Ming Yan, Jianjun Zhang, Qin Xu, Yan Wei, Wantao Chen, Xu Wang

**Affiliations:** 1 Department of Oral and Maxillofacial-Head and Neck Oncology, Shanghai Ninth People’s Hospital, Shanghai Jiao Tong University School of Medicine; College of Stomatology, Shanghai Jiao Tong University; National Center for Stomatology; National Clinical Research Center for Oral Diseases, Shanghai Key Laboratory of Stomatology, Shanghai, 200011 China; 2 Department of Stomatology, Shanghai General Hospital, Shanghai Jiao Tong University School of Medicine, Shanghai, 200080 China; 3 Department of Ophthalmology, Shanghai Xinhua Hospital, Shanghai Jiao Tong University School of Medicine, Shanghai, 200092 China; 4 Department of Ophthalmology, Eye & ENT Hospital, Shanghai Medical College, Fudan University, Shanghai, 200031 China

**Keywords:** Cancer metabolism, Translational research

## Abstract

The histone methyltransferase EZH2 silences gene expression via H3 lysine 27 trimethylation and has been recognized as an important antitumour therapeutic target. However, the clinical application of existing EZH2 inhibitors is not satisfactory for the treatment of solid tumours. To discover novel strategies against head and neck squamous cell carcinoma (HNSCC), we performed genomics, metabolomics and RNA omics studies in HNSCC cells treated with EZH2 inhibitors. It was found that EZH2 inhibitors strongly induced the expression of genes in cholesterol synthesis. Through extensive drug screening we found that inhibition of squalene epoxidase (a key enzyme of endogenous cholesterol synthesis) synergistically increased the squalene content and enhanced the sensitivity of HNSCC cells to EZH2 inhibitors. Our findings provide an experimental and theoretical basis for the development of new combinations of EZH2 inhibitors to treat HNSCC.

## Introduction

As a core component of the polycomb protein family, histone methyltransferase EZH2 and its binding partner proteins form a multisubunit complex, which can specifically catalyse the trimethylation of histone H3 lysine 27 (H3K27me3) and silence target genes in embryonic development and regulate cell proliferation^1^. EZH2 has been found to be highly expressed in many tumours, such as prostate cancer, breast cancer, colorectal cancer, hepatocellular carcinoma, lymphoma and head and neck squamous cell carcinoma (HNSCC)^2^. Overexpression of EZH2 is closely related to tumour progression and poor prognosis^3^. In HNSCC tissues, the upregulation of EZH2 is positively correlated with the overexpression of cyclin D1 and poor clinical prognosis^2^. EZH2 also regulates the STAT3/HOTAIR axis^4^, disrupts differentiation^5^ and promotes oral cancer metastasis by mediating ROS1 oncogene^6^. Currently, several small molecule compounds (EPZ6438, GSK126 and GSK343) have been developed as EZH2 inhibitors^7^. Among them, EPZ6438 has been approved for adults and paediatric patients aged 16 years and older with metastatic or locally advanced epithelioid sarcoma not eligible for complete resection. EPZ6438 has also been approved for adult patients with relapsed or refractory follicular lymphoma whose tumours are positive for an EZH2 mutation as detected by an FDA-approved test and who have received at least 2 prior systemic therapies, and for adult patients with R/R FL who have no satisfactory alternative treatment options^8^. However, the treatment of solid tumours with EZH2 inhibitors is not satisfactory, which limits their clinical application range. Therefore, discovering novel drug combinations of EZH2 inhibitors with other clinical drugs will greatly improve the value of EZH2 inhibitors.

Increasing scientific evidence supports the close relationship between the disorder of cholesterol metabolism and cancer^9^. Dysregulation at different stages of cholesterol metabolism promotes tumour development and resistance to therapeutic drugs^10,11^. On the other hand, some intermediates of cholesterol synthesis and metabolism show obvious antitumour properties. For example, squalene has been reported to block the cell cycle in the G_0_/G_1_ phase^12^. Squalene monooxygenase (SQLE), the key enzyme of the endogenous cholesterol synthesis pathway, catalyses the first oxidation step of cholesterol biosynthesis, converting squalene into 2,3-oxo squalene^13^. Terbinafine and butenafine are used as antifungal agents to inhibit SQLE in fungi^14,15^. However, few studies have reported the antitumour effects of SQLE inhibitors on HNSCC.

In this study, we utilized multiple omics to validate that EZH2 can modulate endogenous cholesterol synthesis and that blocking the endogenous cholesterol synthesis with SQLE inhibitors can largely enhance the sensitivity of HNSCC cells to EZH2 inhibitors.

## Methods and materials

### Cell culture

The human head and neck squamous cell lines HN4, HN6, HN30, CAL27, SCC9 and SCC25 were obtained from the NIH. These cells were cultured in high-glucose medium (L110KJ, Basal Media, China), which contained 10% fetal bovine serum (SA311.02, CellMax, China) 100 U/ml penicillin, and 100 μg/ml streptomycin (C125C5, NCM, China). All cell lines were cultured in incubators with humidified air at 37 °C and 5% CO_2_. Authentication by STR profiling, population doubling time and cell morphology were checked as well to confirm the genotype.

### Lentivirus transfection and screening of stable cell lines

The lentivirus (sh-nc, sh-FDFT1-1, sh-FDFT1-2) were constructed by ZORIN Company (Shanghai, China). According to the manufacturer’s instruction, CAL27 cells were transfected with lentivirus (10^6^ TU/ml) at 40% density. After 72 h in the cell incubator, the cells were cultured in the medium containing 5 μg/ml puromycin for one generation. Then the cells were cultured in the medium containing 10 μg/ml puromycin for two generations. The sequences of sh-nc, sh-FDFT1-1 and sh-FDFT1-2 are showed below: 5ʹ-GCAGUUUCGCAGCUGUUAUTT-3ʹ; 5ʹ-AUAACAGCUGCGAAACUGCTT-3ʹ 5ʹ-GCAGUGCCUGAAUGAAACUUTT-3ʹ; 5ʹ-AAGUUCAUUCAGGCACUGCTT-3ʹ.

### Chemical compounds

The following compounds were purchased from commercial vendors: GSK126 (S7061, Selleck, China), GSK343 (S7164, Selleck, China), EPZ6438 (S7168, Selleck, China), NB-598 (HY-16343C, MCE, China), Atorvastatin (HY-17379, MCE, China), Ro48-8071 (HY-18630A, MCE, China), Terbinafine (HY-17395, MCE, China), Butenafine (HY-17396, MCE, China), Cholesterol (C3045, Sigma, China), Desmosterol (H130206, Aladdin, China), Lathosterol (HY-17395, MCE, China), Squalene (S3626, Sigma, China).

### Cell growth and survival assays

Cells were seeded into 96-well plates at a density of 2000 cells per well, each group has three replicates, and then different inhibitors were added to the plates. Then, 10 μl MTT reagent (Do Jindo, Kumamoto, Japan) was added to 100 μl of culture medium at 72 h. The cells were then incubated at 37 °C for 4 h. After the supernatant was aspirated, 150 μl DMSO was added, and the optical density was measured at 490 nm using a multi-functional microplate reader instrument (Spectra Max i3, Molecular Devices, USA).

### Colony formation

The cells indicated in this study were seeded in 6-well plate (1000 per well) or 24-well plate (300 per well), then cultured with different treatments for 7 days. After fixing with 4% paraformaldehyde for 15 min, the cells were stained with crystal violet for 30 min. Then we use scanners for flat-panel scanning and count the colony numbers by ImageJ.

### Plasmids

The human SQLE Gene ORF cDNA clone expression plasmid was purchased from Sino Biological, China (HG21419-CF), and Pcmv3-c-FLAG was used as the vector.

### qRT-PCR analysis

Total RNA was isolated with the RNAiso Plus kit (TAKARA, China), and reverse transcription of RNA into cDNA using the Prime Script RT Reagent Kit (TAKARA, China). Quantitative PCR was performed using the TB Green Premix Ex Taq reagent kit (TAKARA, China) according to the introduction of ABI Step One Real-Time PCR System (Life Technologies, USA). The sequences of the primers indicated are shown in Supplementary Table S1.

### Immunoblotting analysis

The process of protein extraction and SDS gel electrophoresis is referred to the previous study^16^. The antibodies are listed in Supplementary Table S2.

### Chromatin immunoprecipitation (ChIP) assays

ChIP was performed using the Simple Chip Plus Enzymatic Chromatin IP Kit (Magnetic Beads) (9005S, Cell Signalling Technology, USA) with CAL27 cells, and all procedures were in accordance with the manufacturer’s instructions.

### ChIP-Seq

The procedures of ChIP were performed as the previous study^16^.

In order to remove technical sequences, including adaptors, polymerase chain reaction (PCR) primers or fragments thereof, and quality of bases lower than 20, pass filter data of fastq format were processed by Cutadapt^17^ (version 1.9.1) to be high-quality clean data.

The reference genome sequences and gene model annotation files of relative species are downloaded from genome website, such as UCSC, NCBI and ENSEMBL. The Bowtie2-build is used to index reference genome sequence and clean data are aligned to reference genome via software Bowtie2 (version 2.2.6). MACS2^18^ is used to analyse peaks’ quality control, peaks’ calling and peaks’ annotation. GO and KEGG enrichment analysis were performed as previously described in RNA-Seq analysis.

### RNA interference and transfection

The indicated siRNAs (Supplementary Table S3) were synthesized by GenePharma, China. All siRNAs were transfected into cells using Lipofectamine 3000 (Invitrogen) according to the manufacturer’s instructions.

### Flow cytometric analysis for detecting the cell cycle and cellular apoptosis

CAL27 cells were treated with different drugs for the indicated times. The pretreatment was performed according to the manufacturer’s instructions for PI/RNase Staining Buffer (BD Biosciences, 550825). Then, the cells were analysed on a BD FACSCalibur^TM^ Flow Cytometer (Becton Dickinson, BD Biosciences, USA).

For the apoptosis experiment, the cells treated as described above were washed three times with PBS and centrifuged at 1000 rpm for 3 min. Then, the cells were resuspended in 1× binding buffer and fixed for 30 min. The cells were resuspended with Annexin V and PI dye in binding buffer at a ratio of 1:1:50, placed on ice for 15 min, and then tested on a BD FACSCalibur^TM^ Flow Cytometer (Becton Dickinson, BD Biosciences, USA).

### MS analysis of non-targeted metabolomics

In total, 31 samples of CAL27 cells were collected for non-targeted metabolomics. There were four groups of cells treated with DMSO, EPZ6438, terbinafine or EPZ6438/terbinafine for 72 h. Then 1.5 ml methanol water solution (1:1, V/V) was added to the cell culture dish, and the cell suspension was transferred to the centrifuge tube with cell scrapers. The process of sample processing and computer operation is referred to the published article^19^.

### GC/MS data analysis

Metabolite identification was performed by ChromaTOF software combined with National Institute of Standards and Technology mass spectral libraries (NIST 17), LECO/Fiehn Metabolite mass spectral library (Version 1.00) and our in-house library.

Based on the retention time of a series of fatty acid methyl esters (C6–C28), retention index was calculated by Retention Index Method function in ChromaTOF software^20^. In the meantime, the acquired MS files from GC-TOFMS analysis were processed by ChromaTOF software (v5.50, Leco Co., CA). The data of pretreatment procedures included baseline correction, denoising, time-window splitting, deconvolution, peak filtering and multivariate curve resolution. It is further designed to align and combine peaks from the multiple table files (.csv) obtained from the ChromaTOF software. The resulting three-dimensional data set includes sample information, peak retention time and peak areas. The peak areas were normalized to the area of the internal standards (IS), mean-centred, and then treated by unit variance scaling for further statistical analysis. Finally, IS and any known artificial peaks, such as peaks caused by noise, column bleed and BSTFA derivatization procedure, were removed from the data set^21^.

According to the instructions and Xia and Wishart^22^, the online processing of web page was used to calculate the data expression profile and analyse the function enrichment www.metaboanalyst.ca.

### Detection of squalene monooxygenase activity

The activity of squalene monooxygenase was detected by the kit purchased from Haling Biological Technology Company (GMS50782.1, Shanghai). CAL27 cells were treated with vehicle, EPZ6438, terbinafine and EPZ6438 + terbinafine for 72 h, respectively, according to the manufacture’s instruction. After a series of reactions, the OD values of the standard and the sample was detected at 400 nm by multi-functional microplate reader (Spectra Max i3, Molecular Devices, USA). Finally, the enzyme activity of the samples were calculated according to the instructions.

### Animal experiments

Three-week-old male nude mice were purchased from Shanghai SLAC Laboratory Animal Company and housed in ventilated cages for 1 week. CAL27 cells, sh-*FDFT1* CAL27 cells and sh-nc CAL27 cells (resuspended in DMEM without FBS) were used to establish xenograft models by subcutaneously injecting 100 µl of cells at a concentration of 10^6^ per position in the right and left flanks of each mouse. After 1 week, mice were randomized into four groups based on random number table: vehicle (0.5% CMCNa + 0.1% Tween 80 + ddH_2_O), EPZ6438 (200 mg/kg), terbinafine (60 mg/kg) and EPZ6438 (200 mg/kg) + terbinafine (60 mg/kg). Each group (*N* = 6) received treatment once daily via oral administration. Mouse weight was measured once a day, while tumour volume was measured once every 2 days by callipers. Tumour volume was calculated using the formula *V* = (*L* × *W*^2^)/2. At the end of the study, mice were sacrificed, and tumours were collected and stored at −80 °C until analysis.

### Immunohistochemistry assay and immunofluorescence staining

The paraffin slices were baked in a 65 °C oven for 1 h, soaked in xylene for 10 min, and then soaked in 100% ethanol, 95% ethanol and 75% ethanol for 5 min, respectively. Then, they were boiled in sodium citrate antigen retrieval solution heated to boiling in advance for 30 min to complete the antigen retrieval. A drop of peroxygenase blocking solution was added to each slice, incubated at room temperature for 10 min, and then washed with PBS three times, each time for 10 min. Then, a drop of goat serum was added to each section and incubated at room temperature for 10 min. After removing the serum, 100 μl of primary antibody (1:100) was added to each slide. After incubation at 4 °C overnight, each slice was washed with PBS three times for 10 min each time and then covered with a drop of biotin-labelled secondary antibody and incubated at room temperature for 1 h.

### BODIPY staining

Cell lines were plated in 24-well plates and treated with the indicated pharmacological inhibitors for 48 h. The cells were fixed with 4% paraformaldehyde, washed twice with 1× PBS, and incubated with 1 ml of BODIPY 493/503 (D3922, Thermo Fisher) at 37 °C for 30 min. Then, the cells were washed three times with 1× PBS for 5 min. After DAPI staining for 5 min, the cells were washed three times with 1× PBS for 5 min each time. Images were captured using an inverted fluorescence microscope (AXIO ZEISS, USA).

### RNA-Seq

According to the previous methods^23^, CAL27 cells plated in 10 cm dishes were treated with vehicle or 10 μM EPZ6438 for 72 h. Then, the cells were washed twice with PBS and lysed with an RNAiso Plus kit (TAKARA) according to the manufacturer’s protocol. The cDNA libraries were then constructed for each pooled RNA sample using the VAHTSTM Total RNA-Seq (H/M/R). Differentially expressed mRNAs were then identified through fold change, and *p* values were calculated using *t*-tests. The thresholds set for up- and downregulated genes were fold change ≥ 2.0 and *p* value ≤ 0.05.

### RNA-Seq analysis

The transcriptome sequencing and analysis were conducted by OE Biotech Co., Ltd. (Shanghai, China). Raw data (raw reads) were processed using Trimmomatic^24^. The reads containing ploy-N and the low-quality reads were removed to obtain the clean reads. Then the clean reads were mapped to reference genome using HISAT2^25^.

FPKM^26^ value of each gene was calculated using cufflinks^27^, and the read counts of each gene were obtained by HTSeq-count^28^. DEGs were identified using the DESeq R package function^29^ to estimate Size Factors and nbinom Test. *p* value < 0.05 and fold change > 2 or <0.5 was set as the threshold for significantly differential expression. Hierarchical cluster analysis of DEGs was performed to explore genes expression pattern. GO enrichment and KEGG^30^ pathway enrichment analysis of DEGs were, respectively, performed using R based on the hypergeometric distribution.

### Statistical analysis

The investigators were always blinded to the group allocation during the experiments of the study. Statistical analysis was carried out using or GraphPad Prism 6. Data were presented as the mean ± SD. The correlation was determined by Pearson correlation analysis. One-way analysis of variance (ANOVA) were performed to assess the significance of the differences. Student’s *t*-test was used for pairwise comparisons between groups. A *p* value (two-sided) <0.05 was considered to be statistically significant. Samples sizes were chosen according to the basis of previous publications without prior power analysis.

## Results

### EZH2 inhibitors upregulate the gene expression in the endogenous cholesterol synthesis pathway

**Fig. 1:**
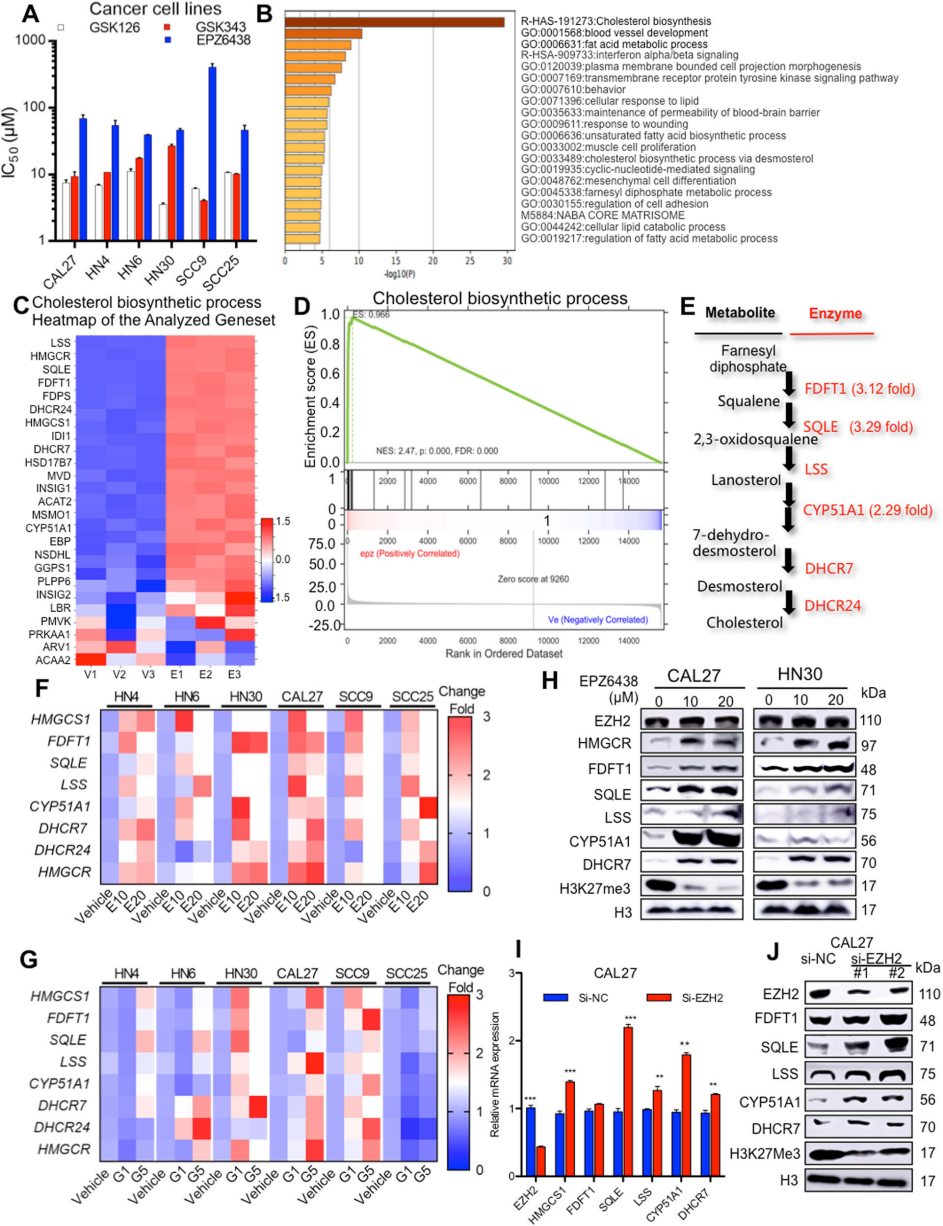
Inhibition of EZH2 promotes the cholesterol synthesis pathway. **A** IC_50_ of three EZH2 inhibitors in different HNSCC cell lines. **B** KEGG enrichment of RNA-Seq data to analyse the significantly 2-fold changed genes in the CAL27 cell line treated with the EPZ6438 compared to the vehicle. **C** The most changed genes in the cholesterol synthesis pathway. **D** GSEA was performed to analyse the expression pattern. **E** Multiple changes in the endogenous enzyme cholesterol synthesis pathway after HNSCC cells were treated with EPZ6438. **F** Real-time PCR assays to detect the EPZ6438-induced expression of cholesterol synthesis enzymes in various HNSCC cell lines. *N* = 3 replicates. E10 (EPZ6438-10 μM), E20 (EPZ6438-20 μM). **G** Real-time PCR assays to detect the GSK126-induced expression of cholesterol synthesis enzymes in various HNSCC cell lines. *N* = 3 replicates. G1 (GSK126-1 μM), G5 (GSK126-5 μM). **H** Immunoblotting analysis to examine the protein levels of cholesterol synthesis enzymes in multiple HNSCC cell lines treated with increasing concentrations of EPZ6438. **I** Real-time PCR assays to detect the expression of target genes in CAL27 cells. *N* = 3 replicates, *t*-test analysis was used to assess the statistical significance, **p < 0.01, ***p < 0.001. **J** Immunoblotting analysis was performed to examine the cholesterol synthesis enzymes after cells were treated with EZH2-specific siRNAs and scrambled siRNA.

To understand the influence of EZH2 inhibitors on the growth of HNSCC, we first tested the sensitivity of different HNSCC cell lines to EZH2 inhibitors. We found that the IC_50_ (half-inhibition concentration) values of most HNSCC cells (HN4, HN6, HN30, CAL27, SCC9 and SCC25) to EZH2 inhibitors (GSK343, GSK126 and EPZ6438) was higher than 10 μM (Fig. 1A). In the RNA-Seq analysis, the EZH2 inhibitors (GSK126 and EPZ6438) had the greatest effect on inducing genes expression of the cholesterol synthesis pathway (Fig. 1B and Supplementary Fig. 1A). The bubble map of pathway enrichment analysis indicated that steroid biosynthesis have the highest enrichment score (Supplementary Fig. 1B, C). The more detailed comparison of enriched genes between the EZH2i group and vehicle group is shown in Fig. 1C and Supplementary Fig. 1D. According to GSEA analysis of the RNA-Seq data, we found that genes in cholesterol biosynthesis process were statistically enriched (false discovery rate [FDR] *q* < 0.05) in the cells treated with EZH2i than in the vehicle group (Fig. 1D and Supplementary Fig. 1E). The expression of SQLE was increased over 3-fold (Fig. 1E and Supplementary Fig. 1F). Next, we selected three small molecular compounds to incubate different HNSCC cells. All the inhibitors significantly upregulated the mRNA level of endogenous cholesterol synthesis-related enzymes, including *HMGCS1*, *FDFT1*, *SQLE*, *LSS*, *CYP51A1*, *DHCR7*, *DHCR24* and *HMGCR* (Fig. 1F, G and Supplementary Fig. 1G). The protein levels were also significantly increased (Fig. 1H). Moreover, the mRNA and protein levels of the target genes were further verified in the cells that were knocked down of EZH2 by specific siRNAs compared to the scramble siRNA (Fig. 1I, J). These data suggest that the inhibition of EZH2 significantly upregulates the gene expression of the endogenous cholesterol synthesis pathway in HNSCC cells.

### EZH2 inhibitor promotes the cholesterol synthesis pathway by H3K27me3 modification

**Fig. 2:**
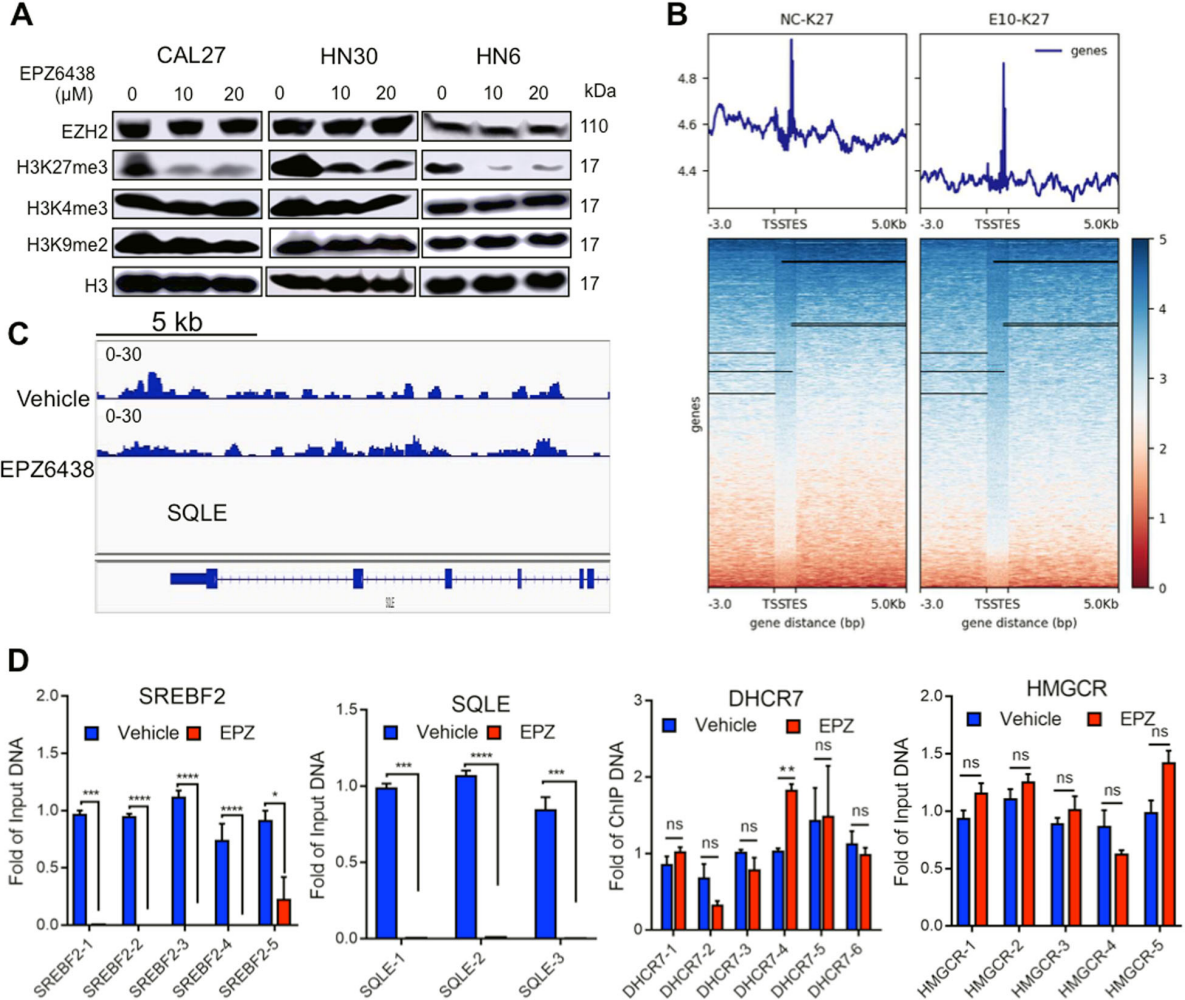
EZH2 regulates cholesterol synthesis through H3K27me3. **A** Immunoblotting analysis to examine the methylation modification of different histone residues by the EZH2 inhibitor EPZ6438. **B** Heatmaps of H3K27me3 ChIP-Seq signals at the TSS in vehicle- and EPZ6438-treated CAL27 cells. **C** Genome browser view of normalized ChIP-seq signals of H3K27me3 at the *SQLE* locus in vehicle and EPZ6438-treated CAL27 cells. **D** ChIP-PCR analysis to examine the level of H3K27me3 modification in the promoter regions of different target genes after CAL27 cells were treated with vehicle or EPZ6438. *N* = 3 replicates, *t*-test analysis was used to assess the statistical significance, *p < 0.05, **p < 0.01, ***p < 0.001, ****p < 0.0001.

To examine whether EZH2 can specifically regulate the expression of the cholesterol synthesis pathway by modulating H3K27me3 levels, HNSCC cells were incubated with the EZH2-specific inhibitor EPZ6438. EPZ6438 selectively reduced H3K27me3, rather than other modifications (Fig. 2A). Next, we used H3K27me3 antibody to enrich target genes in the ChIP assay (Fig. 2B). Compared to the vehicle control group, H3K27me3 modification were enriched at the loci of SQLE promoter and other genes in cholesterol synthesis (Fig. 2C and Supplementary Fig. 2A, B). The H3K27me3 levels of SREBF2 and SQLE decreased significantly after EZH2 inhibitor treatment, but the levels of other genes involved in cholesterol metabolism did not change significantly (Fig. 2D). These data suggest that EZH2 regulates the expression of related metabolic enzymes in the cholesterol synthesis pathway at least partially through H3K27me3.

### Inhibiting the cholesterol metabolic pathway enhances the sensitivity of HNSCC to EZH2 inhibitors

**Fig. 3:**
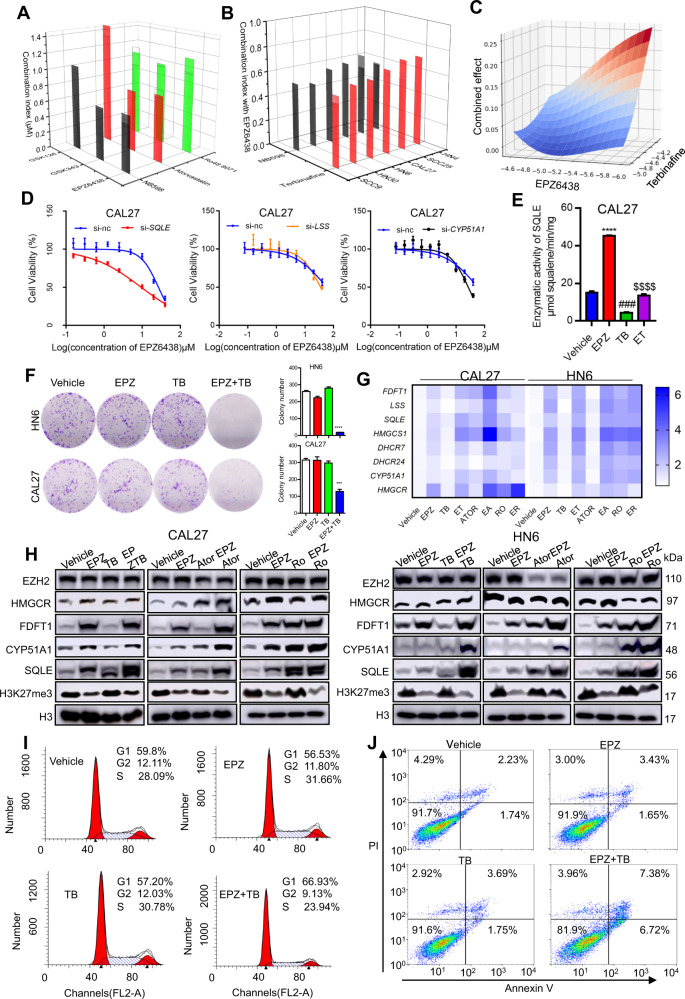
Dual pharmacological inhibition of EZH2 and SQLE synergistically decreases the proliferation of HNSCC. **A** The combination index (CI) of EZH2 inhibitors and key enzyme inhibitors against cholesterol synthesis in CAL27 cells. *N* = 3 replicates. **B** The viability of CAL27 cells was detected by an MTT assay after EPZ6438 incubation and silencing of the corresponding gene with siRNAs. **C** The CI value of EPZ6438 combined with NB-598 or terbinafine in different cells. **D** The Bliss model was used to calculate the combination effect of EPZ6438 and terbinafine. **E** The enzyme activity of SQLE in the CAL27 cell with indicated treatment. *N* = 3 replicates, one-way ANOVA analysis was used to assess the statistical significance, ****p < 0.0001, EPZ vs vehicle; ^###^*p* < 0.001, TB vs vehicle; ^$$$$^*p* < 0.0001, ET vs EPZ. **F** Representative images and quantitative analysis of colony formation to indicate the potent synergistic anti-proliferative effect of EPZ6438 and terbinafine on HN6 and CAL27 cells. *N* = 3 replicates, one-way ANOVA analysis was used to assess the statistical significance, ***p < 0.001, ****p < 0.0001. **G** Real-time PCR detection to examine the expression of cholesterol synthesis-related genes in CAL27 and HN6 cell lines with different treatment. EPZ (single EPZ6438), TB (single terbinafine), ET (EPZ6438 + terbinafine), ATOR (atorvastatin), EA (EPZ6438 + atorvastatin), RO (Ro-488071), ER (EPZ6438 + Ro-488071), *N* = 3 replicates. **H** Immunoblotting assays to detect the protein levels of cholesterol synthesis-related genes in CAL27 and HN6 cell lines. **I** Flow cytometry examination to detect the cell cycle of CAL27 cells with different treatments for 48 h. **J** Flow cytometry examination to detect the apoptosis of CAL27 cells with different treatments for 72 h.

Next, we incubated CAL27 cells with the combination of EZH2 inhibitors (GSK126, GSK343 and EPZ6438) and inhibitors of different key enzymes in the cholesterol synthesis (the SQLE inhibitor NB-598, the HMGCR inhibitor atorvastatin and the LSS inhibitor Ro48-8071). All the combination indexes (CI) of EZH2 inhibitors plus NB-598 were lower than those of other formulas and indicated a potent synergistic effect (CI < 0.6) (Fig. 3A). Furthermore, we analysed the combination effect of EPZ6438 and terbinafine through orthogonal test. EPZ6438 and terbinafine have a strong inhibitory effect on CAL27 cells (Supplementary Fig. 3B). Except for CAL27 cells, terbinafine and EPZ6438 indicated potent synergistic effects on a variety of cell lines (HN4, HN6, HN30, CAL27, SCC9 and SCC25) (Fig. 3B). Another evaluation in the Bliss model indicated that the combination scores of EPZ6438 and terbinafine are lower than 0.3 (Fig. 3C). The cholesterol metabolizing enzyme genes such as *SQLE*, *FDFT1*, *LSS*, *CYP51A1*, *DHCR7* and *HMGCR* were silenced with siRNAs and verified efficiency with real-time PCR assays (Supplementary Fig. 3A). The sensitivity of HNSCC cells to EPZ6438 increased significantly when the expression of SQLE was silenced, rather than other genes (Fig. 3D and Supplementary Fig. 3C). EPZ6438 substantially enhances the enzymatic activity of SQLE in CAL27 cells, which was completely opposite to terbinafine. There was no significant difference between the combination group and the vehicle group (Fig. 3E). The colony number in the combination group was significantly lower than that in the control group (Fig. 3F). And EPZ6438 treatment also synergistically inhibited cell viability with the specific siRNA against SQLE (Supplementary Fig. 3D). Compared with the vehicle group, both mRNA and protein expressions of the cholesterol synthesis enzymes were upregulated in the combination group in HN6 and CAL27 cell lines (Fig. 3G, H). According to the results of flow cytometry, the CAL27 and HN6 cells were significantly blocked in G_1_ phase after the treatment with the combination (Fig. 3I and Supplementary Fig. 3E). Meanwhile, the combination also promoted the apoptosis of CAL27 and HN6 cells (Fig. 3J and Supplementary Fig. 3F). These data suggest that inhibiting SQLE and EZH2 could arrest the tumour cells in G_1_ phase and reduce the growth of tumour cells.

### Accumulation of squalene in HNSCC cells inhibits cell growth

**Fig. 4:**
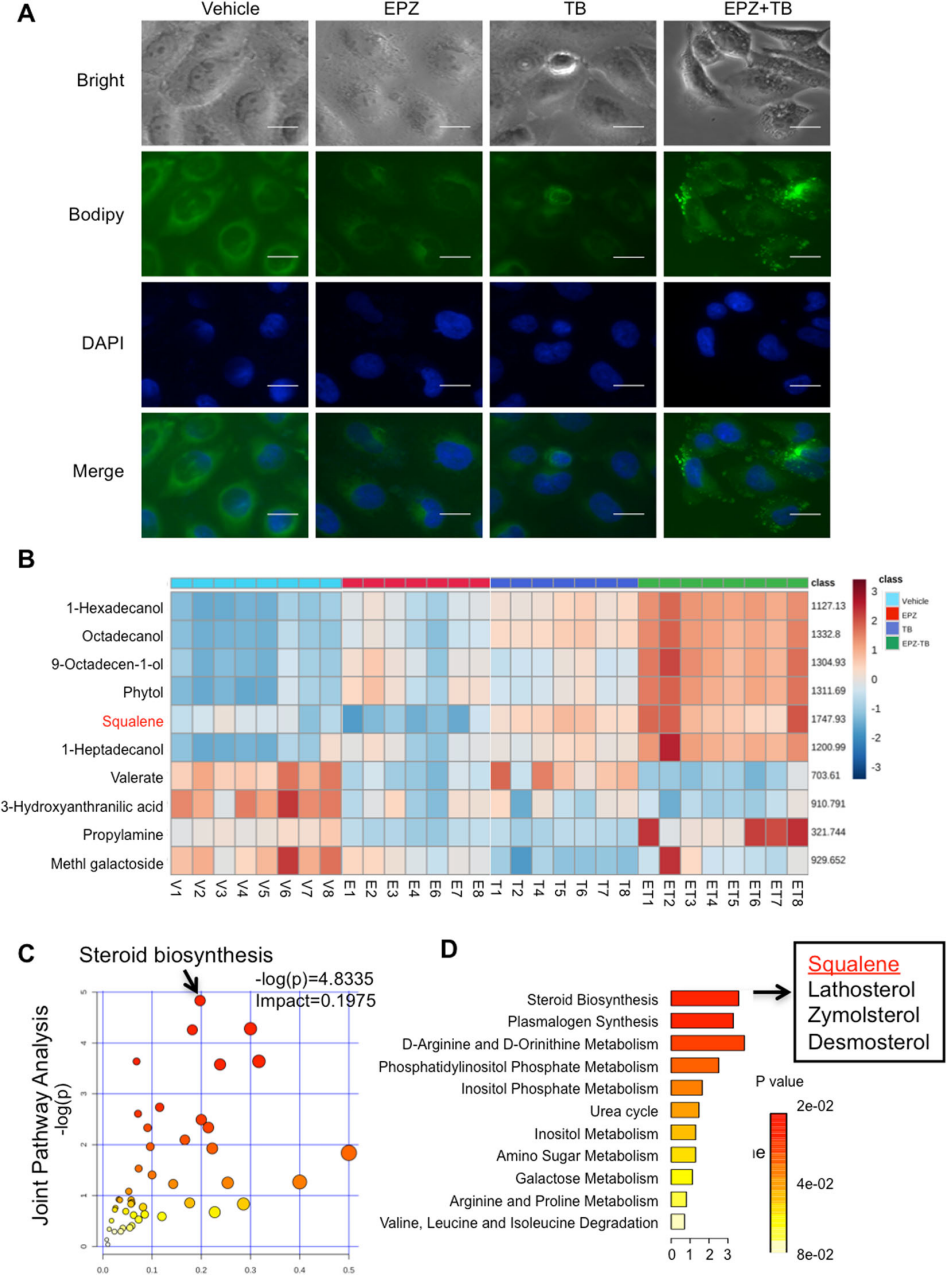
Double inhibition of EZH2 and SQLE promotes lipid accumulation in HNSCC cells. **A** Morphological changes and BODIPY staining of neutral lipids in HN6 cells in different treatment groups. Cells were treated with vehicle, EPZ6438, terbinafine or the combination for 48 h. Bar = 100 μm. **B** Heatmap of the top 10 metabolites with significant changes among 289 metabolites detected by GC/MS/MS in CAL27 cells after treatment for 72 h. *N* ≥ 7 replicates. **C** The combined analysis of gene expression and metabolism profile. The sterol biosynthesis pathway was enriched with the highest score by the combination of EZH2 inhibitor and SQLE inhibitor. **D** Squalene is one of the four changed metabolites involved in sterol biosynthesis.

Upon morphological examination, the tumour cells turned slender by the co-treatment with EPZ6438 and terbinafine contained many vacuoles in the cellular plasma (Fig. 4A). As indicated in the BODIPY staining assay, more lipid drops accumulated in the cellular plasma of combination group than those of other groups (Fig. 4A). To examine the possible accumulated lipids, the comparative detection of 297 metabolites were performed with mass spectrometry. Although some lipid metabolites of EPZ6438 or TB-treated cells were slightly elevated, the content of lipid metabolites in the drug combination group was different from that in the control group (Fig. 4B). The top 10 changed metabolites were enriched. In particular, the contents of 1-hexadecanol, octadecanol, 9-octadecen-1-ol, phytol, squalene and 1-heptadecanol increased after drug combination. Through joint pathway analysis with RNA-Seq, the steroid biosynthesis was the pathway with highest impact score in cells treated with the combination compared to the vehicle group (Fig. 4C). Four metabolic substances accumulated in the co-treatment group, including squalene, lathosterol, zymosterol and desmosterol (Fig. 4D).

**Fig. 5:**
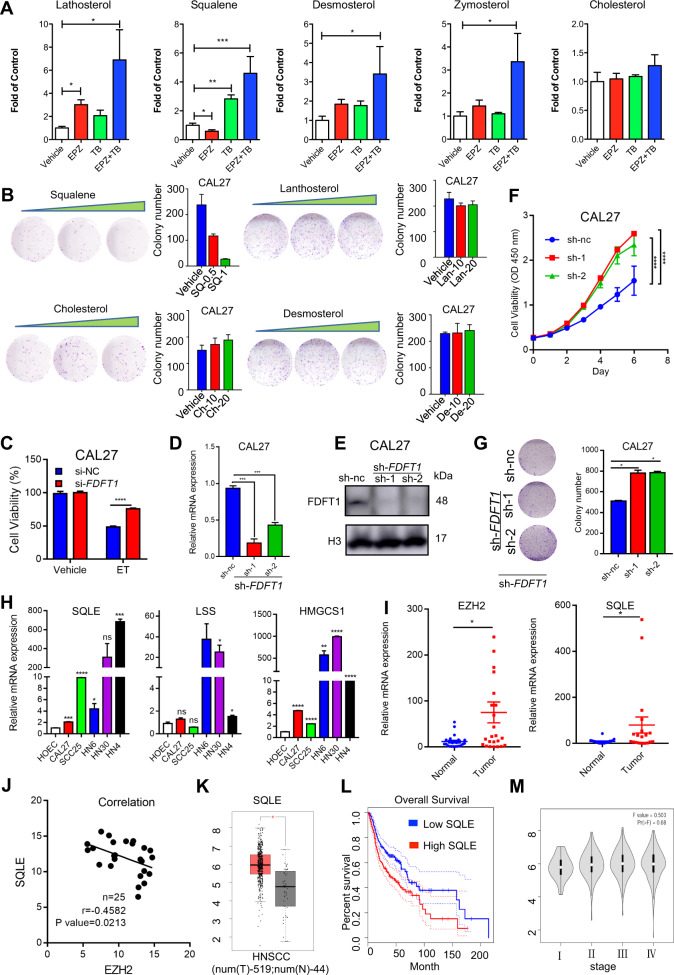
Squalene accumulation potently inhibits the proliferation of HNSCC cells. **A** Changes in four cholesterol metabolites in different treatment groups detected by GC/MS/MS analysis. *N* ≥ 7 replicates, one-way ANOVA analysis was used to assess the statistical significance, *p < 0.05, **p < 0.01, ***p < 0.001. **B** Colony formation of CAL27 cells was detected after the cholesterol metabolites were supplemented in cell culture medium. **C** The viability of CAL27 cells was detected with an MTT assay after blocking endogenous squalene production by knockdown of FDFT1 with specific siRNAs. **D** The real-time PCR assays to detect the protein levels of FDFT1 in CAL27 cells treated with sh-NC or sh-FDFT1 lentivirus. **E** The immunoblotting assays to detect the protein levels of FDFT1 in CAL27 cells treated with sh-NC or sh-FDFT1 lentivirus. **F** The cell proliferation of CAL27 cells treated with sh-NC or sh-FDFT1 lentivirus. **G** The colony formation of CAL27 cells treated with sh-NC or sh-FDFT1 lentivirus. **H** Expression of cholesterol synthesis-related enzymes in HNSCC cell lines and oral mucosa epithelial cells. **I** Expression of SQLE and EZH2 in HNSCC (*N* = 25). **J** There was a negative correlation between the expression of EZH2 and SQLE in HNSCC (*N* = 25). **K** Expression of SQLE in HNSCC and normal tissues in TCGA database. **L**, **M** Relationship between expression level of SQLE in TCGA database and survival rate and clinical stage.

According to the mass spectrometry analysis, the single EPZ6438 treatment significantly decreased the cellular content of squalene, while the co-treatment of EPZ6438 and terbinafine accumulated squalene content (Fig. 5A). Although lanosterol and 24-dehydrocholesterol were also accumulated in the combination group, there was no significant change in the cholesterol content among the different groups (Fig. 5A). To verify the effect of metabolites on HNSCC cells, we supplemented the cholesterol, squalene, lanosterol and desmosterol to the culture medium. The number of colony formation in squalene-treated group was dose-dependently reduced, while other metabolites had no obvious effects on colony formation (Fig. 5B). After the squalene synthase FDFT1 was silenced by specific siRNAs, the combination of EPZ6438 and terbinafine inhibited less cell viability in the si-FDFT1 cells than in the si-NC cells (Fig. 5C). To further examine the role of FDFT1, the CAL27 cells were infected with sh-FDFT1 lentivirus and established as stable cell lines (Fig. 5D, E). The proliferation of sh-FDFT1 cells was much higher than that of the sh-NC cells (Fig. 5F, G), suggesting that the production of squalene is negatively related to the cell proliferation.

The expression level of SQLE in HNSCC cell lines was significantly higher than that in normal human oral epithelial cells (HOEC) (Fig. 5H and Supplementary Fig. 4). Although the mRNA expression of EZH2 and SQLE were highly expressed in HNSCC samples (Fig. 5I), the expression of EZH2 was negatively correlated with the expression of SQLE in HNSCC tissues (Fig. 5J). Moreover, the expression of SQLE in HNSCC tissues of TCGA library was significantly higher than that in normal tissues (Fig. 5K). The expression level of SQLE mRNA was negatively correlated with the survival of patients, but not with the tumour stage (Fig. 5L, M).

The above results indicate that the simultaneous inhibition with EZH2 and terbinafine cause accumulation of squalene and inhibition of tumour cell growth.

### The combination of EZH2 inhibitor and SQLE inhibitor reduces HNSCC growth in vivo

**Fig. 6:**
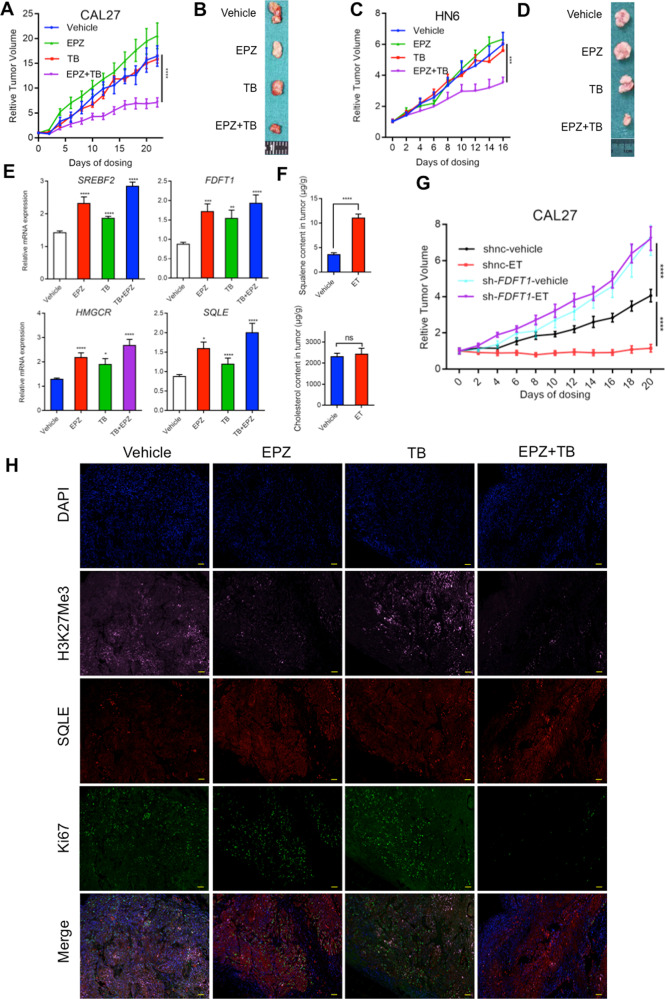
Double inhibition of EZH2 and SQLE decreases HNSCC tumour growth in vivo. **A** Relative tumour (CAL27 cells) volume showed that EZH2 and SQLE inhibitors significantly inhibited tumour growth. *N* = 6 replicates, two-way ANOVA analysis was used to assess the statistical significance, ***p < 0.001, ****p < 0.0001. **B** Representative image of the tumour (CAL27 cells). **C** Relative tumour (HN6 cells) volume showed that EZH2 and SQLE inhibitors significantly inhibited tumour growth. *N* = 6 replicates, ***p < 0.001, ****p < 0.0001. **D** Representative image of the tumour (HN6 cells). **E** Detection of cholesterol metabolizing enzyme-related genes in tumour tissues by real-time PCR. *N* = 3 replicates, one-way ANOVA analysis was used to assess the statistical significance, *p < 0.05, **p < 0.01, ***p < 0.001, ****p < 0.0001 vs vehicle. **F** Quantitative concentrations of squalene in vehicle or ET combination-treated CAL27 tumours. *N* = 3 replicates, two-way ANOVA analysis was used to assess the statistical significance, ****p < 0.0001. **G** Relative tumour volume showed that combination of EZH2 and SQLE inhibitors failed to inhibit tumour growth of sh-FDFT1-treated CAL27 xenograft compared to the sh-NC group. **H** The expression levels of SQLE, H3K27me3 and Ki67 in tumour tissues of different treatment groups were detected by immunofluorescence staining.

**Fig. 7:**
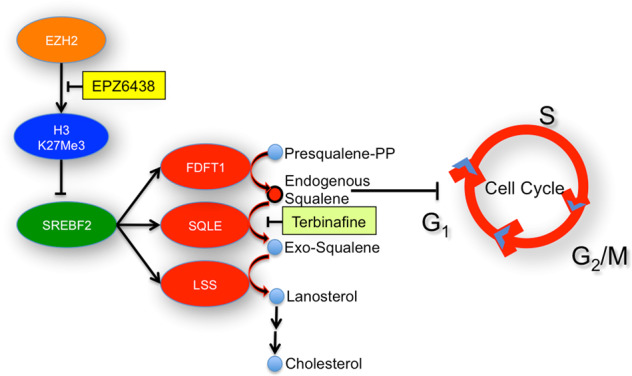
Simultaneously targeting of EZH2 and SQLE significantly inhibits the tumour growth of HNSCC. A schematic illustration of the proposed model in which EZH2 inhibition sensitizes SQLE inhibitors to inhibit HNSCC growth.

Next, we established the xenograft models with CAL27 and HN6 cells to examine the effect of the combination in vivo. The co-treatment with EPZ6438 and terbinafine significantly inhibited tumour growth compared with the vehicle group (Fig. 6A–D and Supplementary Fig. 5A, B). The co-treated tumour weight was significantly lower than that of the vehicle group (Supplementary Fig. 5C). There was no statistical significance in the weight change of mice between the four groups (Supplementary Fig. 5D). Based on the real-time PCR detection in tumour tissue, EPZ6438 significantly upregulated the expression of cholesterol synthesis-related genes, but the combination increased the expressions much higher than any other groups (Fig. 6D and Supplementary Fig. 5E). Meanwhile, in the quantitative analysis of mass spectrometry, the content of squalene in the tumour tissues of combination group were significantly higher than that in the vehicle group (Fig. 6E). Furthermore, we detected the subcutaneous tumorigenesis using sh-FDFT1 and sh-NC CAL27 cells. In the tumours of sh-FDFT1 group, the combination of EPZ6438 and terbinafine failed to inhibit tumour growth, indicating that the necessity of squalene production (Fig. 6F and Supplementary Fig. 5F). In the images of tumour tissues stained by polychromatic immunofluorescence, H3K27me3 levels were reduced by EPZ6438, while the expression of SQLE increased significantly upon EPZ6438, terbinafine and combination treatment in the identical area of tumour tissue. In addition, compared with the vehicle group, EPZ6438 combined with terbinafine significantly reduced Ki67 signal in the same area (Fig. 6G and Supplementary Fig. 5G). Together, these data suggest that simultaneously targeting of EZH2 and SQLE could significantly inhibit the tumour growth of HNSCC. Together, these data suggest that simultaneously targeting of EZH2 and SQLE could significantly inhibit the tumour growth of HNSCC (Fig. 7).

## Discussion

EZH2 has been widely studied as an important antitumor target. In most solid tumours, EZH2 is present in an overexpressed wild-type form that bears H3K27me catalysing activity. Therefore, more research on the combination of EZH2 inhibitors and tumour therapy has emerged. It has been reported that EZH2i and BRD4i regimens or triple combinations that include MAPK inhibitors can be used for EZH2-positive tumour patients^31^. EZH2 inhibitors and PARP inhibitors can synergistically inhibit the growth of ovarian cancer^32^. It remains unclear, however, whether overexpressing EZH2 could also functionally result in growth dependency of endogenous cholesterol synthesis. Insight into this problem may expand the therapeutic benefits of EZH2 inhibitors to a broad spectrum of solid tumours. In the current study, we discovered a novel combination strategy of EZH2 inhibitors based on cholesterol metabolism in HNSCC.

Cholesterol is a unique lipid that is essential in the structure of bilayer phospholipids, so it plays an important role in biofilm formation, cell proliferation and cell differentiation. In addition, cholesterol regulates the functions of different receptors, participates in molecular and vesicular transport and is also the precursor of lipid-soluble vitamins, bile acids and steroid hormones^11^. Tumour cells support cell survival and rapid proliferation by reprogramming the cholesterol metabolism pathway^33^. Under normal circumstances, to maintain the appropriate cholesterol balance in cells, multiple pathways strictly control cholesterol homeostasis^34^. However, there are a few studies on the regulation of cholesterol metabolism at the epigenetic level of cells. Our data suggest that the transcription factor SREBF2 and its target gene SQLE are directly regulated by EZH2-mediated H3K27me3 modification in HNSCC cells.

SQLE is highly expressed in the liver, nervous tissue, digestive tract and skin^35^. High expression of SQLE and high copy number of the *SQLE* gene are closely related to poor prognosis of many tumours^36^. Like almost all genes encoding cholesterol synthase, SQLE is a target gene of SREBPs. SREBPs are activated when the concentration of sterol in cells is reduced, and the binding of SREBPs with the SRE sequence leads to the transcription of the *SQLE* gene^37^. The high copy number (CN) of the *SQLE* gene has been reported in a variety of tumours, including breast cancer, prostate cancer, lung cancer and pancreatic cancer^38^. Knockdown of *SQLE* or the use of SQLE inhibitors can inhibit the growth of breast cancer cells and NAFLD-HCC^39^, and the copy number of the *SQLE* gene in tumour cells is positively correlated with the sensitivity of SQLE inhibitors. Further studies should examine the relationship between high copy number and the efficacy of combination with EZH2i plus SQLEi.

The EPZ6438 induced the mRNA expression of HMGCS, SQLE and LSS through decreasing the H3K27me3 at the promoter region of these genes as an epigenetic modification. We observed the single TB treatment also increased the SQLE mRNA. Some enzyme inhibitors could induce the mRNA of the enzyme through the transcript factors at the transcriptional regulation^40^. TB is the activity inhibitor of SQLE. Therefore, we detected the activity of SQLE, and concluded that the effect of terbinafine was to inhibit the enzyme activity of SQLE. When the two drugs are used in combination, the expression of HMGCS, SQLE and LSS is increased above the single agents alone, indicating that mRNA expressions are separately modulated by epigenetic and transcriptional mechanism.

SQLE inhibitors have been extensively investigated for a long time for their antifungal properties^41^. Notably, several studies conducted in recent years demonstrated that the fungal SE inhibitor terbinafine decreased cell number and viability in various cultured human malignant cells in a dose-dependent manner, arresting the cell cycle at the G_0_/G_1_ transition^42–44^. Accordingly, we chose terbinafine as the agent to inhibit SQLE in HNSCC cells for potential further clinical research in combination with EPZ6438. The IC_50_ values of terbinafine for mammalian SQLE are an order of magnitude higher than that for the fungal enzyme. Hence, during the anti-cancer treatment, further potent SQLE inhibitors will produce more synergistic effects than terbinafine. Currently, there are several NB-598 derivatives targeting SQLE in human cancer cells^45,46^. Our study will provide a potential option for combined treatment with terbinafine and EPZ6438 in HNSCC patients.

The core of the study is to examine the certain relationship between EZH2 and SQLE, and the combined drug treatment has produced a certain anti-cancer effect. The anti-cancer effect of ET combination is dependent on the presence of endogenous expression of FDFT1, while the FDFT1 is the key enzyme to produce squalene. However, still a lot of work should be performed in the further studies to discover the detailed mechanism. We still could not exclude that other potential pathways are involved in the anti-cancer effect of ET combination.

In conclusion, EZH2 reprogramed the endogenous cholesterol synthesis via modulating H3K27me3 levels; dual inhibition of EZH2 and SQLE caused cellular accumulation of squalene and synergistically inhibited HNSCC growth. Our study may provide novel insight showing that solid tumours are not sensitive to EZH2 inhibitors.

## Supplementary information

Supplementary Files.
