# Supplementary material for: Targeting epigenetic modulation of cholesterol synthesis as a therapeutic strategy for head and neck squamous cell carcinoma
Source: Cell Death Dis. 2021 May 13;12(5):482. doi: 10.1038/s41419-021-03760-2 (PMC8119982; doi:10.1038/s41419-021-03760-2)
Supplement: Supplementary file 1 — Supplementary Files. [file 41419_2021_3760_MOESM1_ESM.doc]

***Supplementary Figures***

***
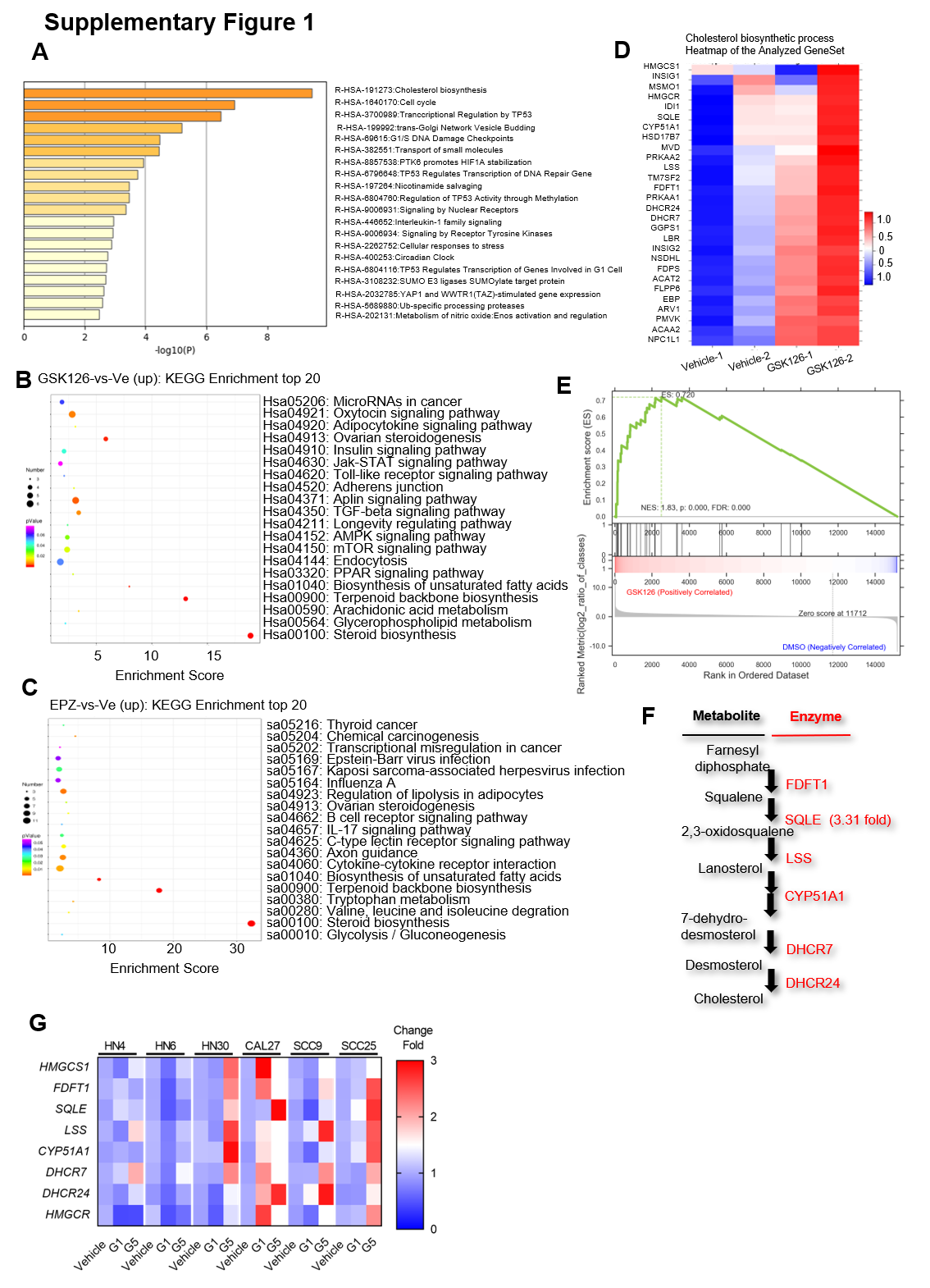
***

Supplementary Figure 1

A. KEGG enrichment of RNA-Seq data to analyze the significantly 2-fold changed genes in the CAL27 cell line treated with the GSK126 compared to the vehicle.

B. The bubble map of pathway enrichment analysis for differentially expressed genes in CAL27 treated with GSK126.

C. The bubble map of pathway enrichment analysis for differentially expressed genes in CAL27 treated with EPZ6438.

D. The most changed genes in the cholesterol synthesis pathway in the CAL27 cell line treated with the GSK126 compared to the vehicle.

E. GSEA was performed according to the FDR q value, and the darkest blue represents q R 0.1 or N/A. CAL27 cell line treated with the GSK126 compared to the vehicle.

F. Multiple changes in the endogenous enzyme cholesterol synthesis pathway after HNSCC cells were treated with EPZ6438.

G. Real time PCR assays to detect the GSK343-induced expression of cholesterol synthesis enzyme in various HNSCC cell lines. N=3


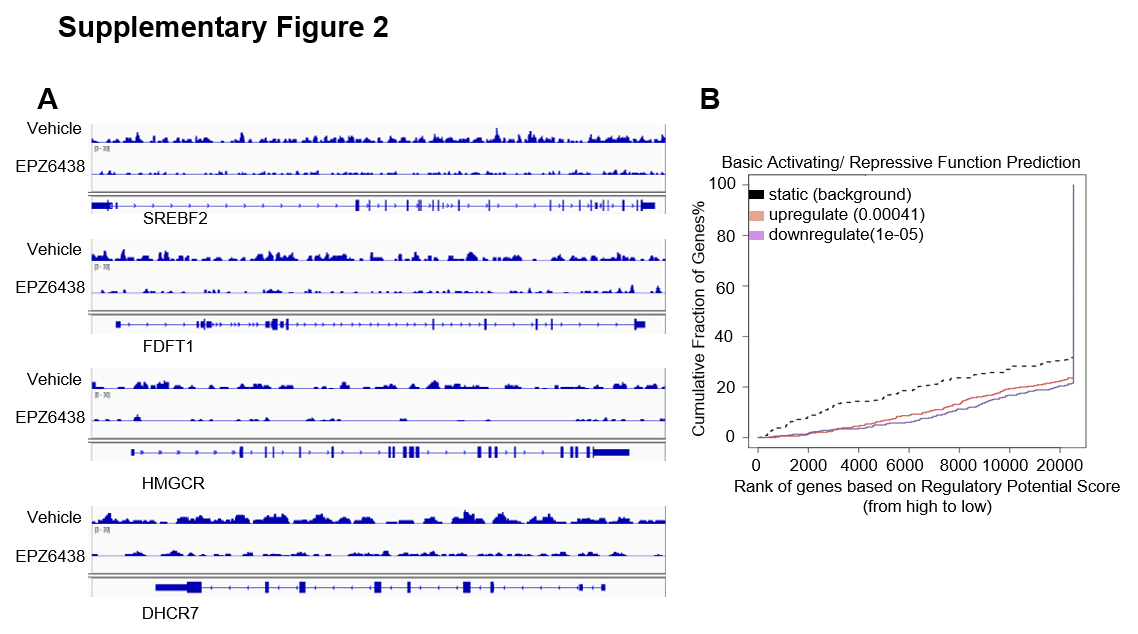


Supplementary Figure 2

A. Genome browser view of normalized ChIP-seq signals of H3K27me3 at the different genes locus in vehicle and EPZ6438-treated CAL27 cells.

B. Combined analysis of RNA-seq and ChIP-seq data.


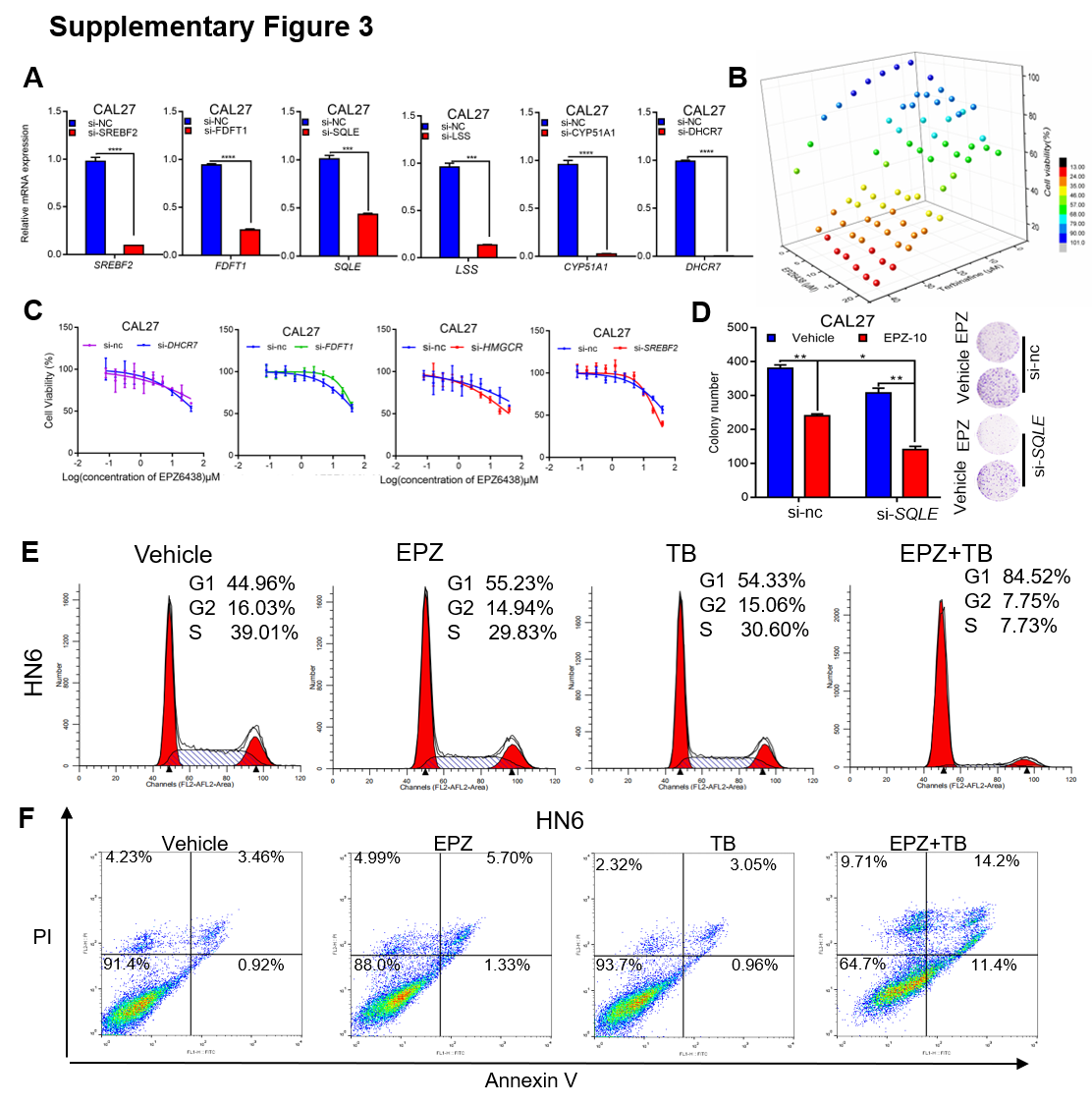


Supplementary Figure 3

A. Detection of siRNA interference efficiency by Real time PCR.N=3. Statistical analysis was performed using t-test, *****P* < 0.0001

B. Effects of EPZ6438 and terbinafine on the proliferation of CAL27 cells by orthogonal test.

C. The cell viability of CAL27 cells was detected by MTT after EPZ6438 incubation and silencing the corresponding gene with siRNAs.

D. Detection of synergistic effect of EPZ6438 and SQLE specific siRNA on HN6 cells by colony formation assays.

E. Flow cytometry examination to detect the cell cycle of HN6 cells with different treatments for 48 hours.

F. Flow cytometry examination to detect the cell apoptosis of HN6 cells with different treatments for 72 hours.


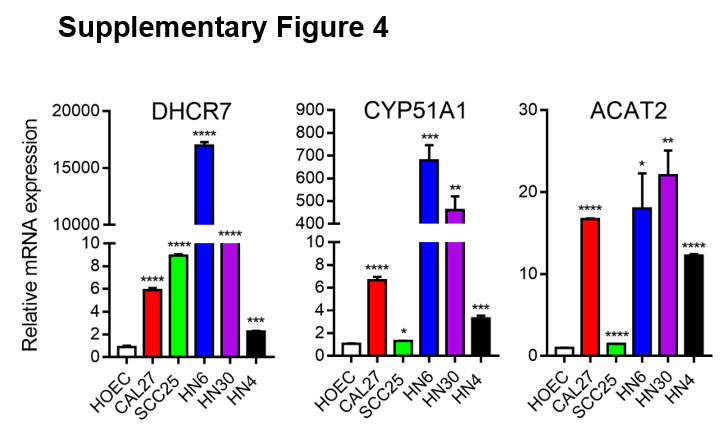


Supplementary Figure 4

Expression of cholesterol synthesis related enzymes in HNSCC cell lines and oral mucosa epithelial cells. Statistical analysis was performed using t-test, *p < 0.05, **p < 0.01, ***p < 0.001, ****p＜0.0001.


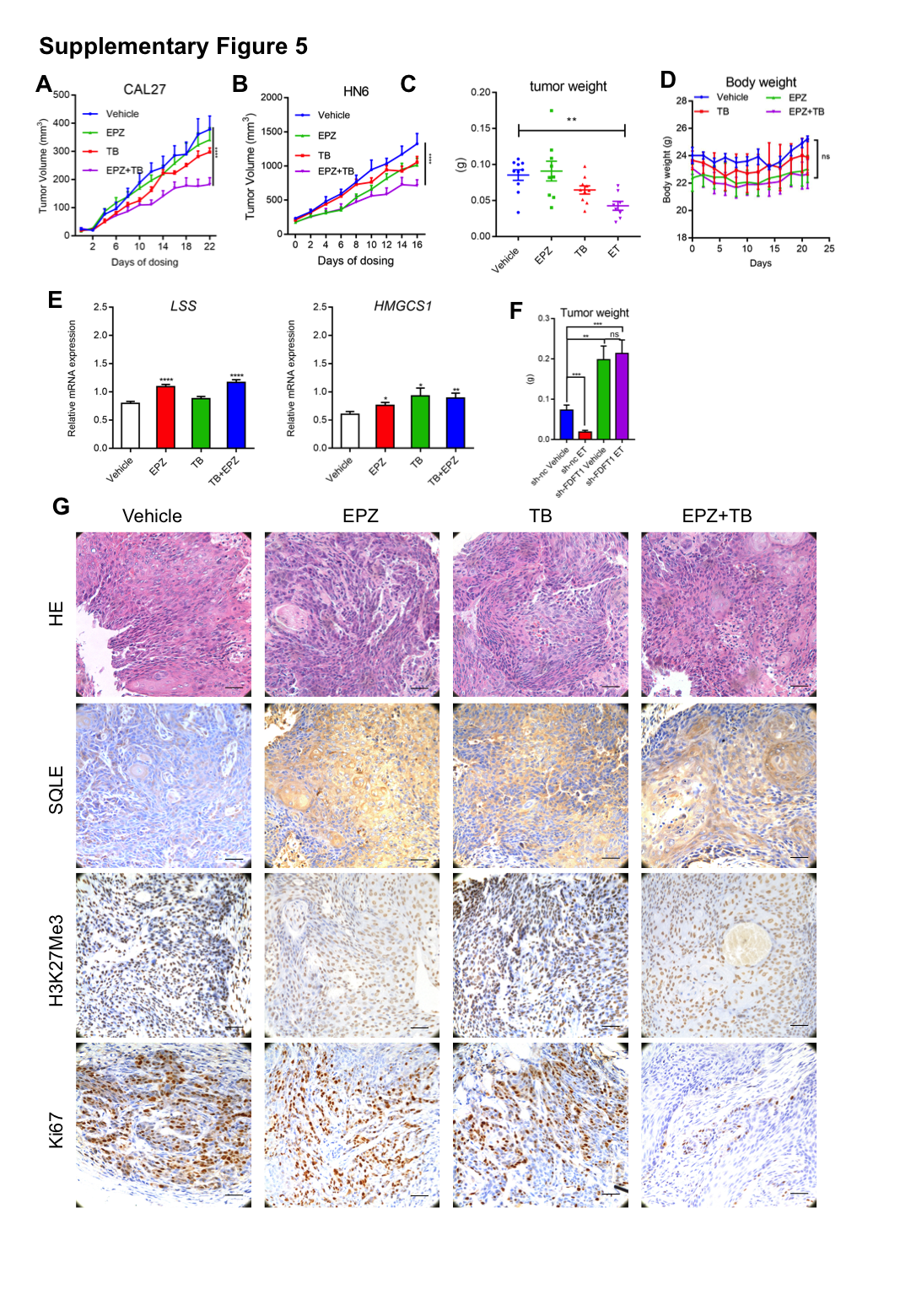


Supplementary Figure 5

A. Tumor (CAL27 cell line) volume showed that EZH2 and SQLE inhibitors significantly inhibited tumor growth. N≥8 replicates, Two way ANOVA analysis was used to assess the statistical significance.****p＜0.0001.

B. Tumor (HN6 cell line) volume showed that EZH2 and SQLE inhibitors significantly inhibited tumor growth. N≥8 replicates, Two way ANOVA analysis was used to assess the statistical significance. ****p＜0.0001.

C. In the xenografts of CAL27 cells subcutaneously transplanted into tumors, the tumor weight of the co-treatment groups was lower than that in solvent control group.

D. In the xenografts of CAL27 cells subcutaneously transplanted into tumours, there was no significant difference in body weight between the treatment groups and the solvent control group.

E. Detection of cholesterol metabolizing enzyme-related genes in tumor tissues by real-time PCR. N=3, Statistical analysis was performed using One way ANOVA method, *p < 0.05, **p < 0.01.

F. In the xenografts of sh-*FDFT1* cells subcutaneously transplanted into tumors, there was no significant difference in tumor weight between the co-treatment groups and the solvent control group. N=12, Two way ANOVA analysis was used to assess the statistical significance.**p < 0.01, ***p < 0.001, ****p＜0.0001.

G. HE staining of tumour tissue sections in each group, and the expression levels of SQLE, H3K27me3 and Ki67 in tumour tissues of different treatment groups were detected by immunohistochemical staining.

Supplementary Table 1

The sequences of primers for detecting target genes by qRT-PCR.

| Primers | Forward | Reverse |
| --- | --- | --- |
| EZH2 | GGACCACAGTGTTACCAGCAT | GTGGGGTCTTTATCCGCTCAG |
| SREBF2 | GGCTGAAGAATAGGAGTTGCC | AACGGTCATTCACCCAGGTC |
| HMGCS1 | CCCAGCCAATGCTTCAGGAAT | AAGCCCACGTTTATCAGCTTC |
| FDFT1 | CCACCCCGAAGAGTTCTACAA | TGCGACTGGTCTGATTGAGATA |
| SQLE | TGACAATTCTCATCTGAGGTCCA | CAGGGATACCCTTTAGCAGTTTT |
| LSS | GCACTGGACGGGTGATTATGG | TCTCTTCTCTGTATCCGGCTG |
| CYP51A1 | GAAACGCAGACAGTCTCAAGA | ACGCCCATCCTTGTATGTAGC |
| HMGCR | TGATTGACCTTTCCAGAGCAAG | CTAAAATTGCCATTCCACGAGC |
| ACAT2 | CCCAGCCAATGCTTCAGGAAT | AAGCCCACGTTTATCAGCTTC |
| DHCR7 | GCAGGGGTTGTGAACAAGTAT | GAGACGGCATAGCCAAGGAT |
| 18sRNA | CAGCCACCCGAGATTGAGCA | TAGTAGCGACGGGCGGGTGT |

Supplementary Table 2

The information of antibodies indicated above.

| Antibodies | Source | Identifier |
| --- | --- | --- |
| EZH2 | ABclonal technology | Cat#A16846 |
| SREBF2 | ABclonal technology | Cat#A13049 |
| FDFT1 | ABclonal technology | Cat#A6229 |
| SQLE | ABclonal technology | Cat#A2428 |
| LSS | ABclonal technology | Cat#A6930 |
| CYP51A1 | ABclonal technology | Cat#A13485 |
| HMGCR | ABclonal technology | Cat#A19063 |
| DHCR7 | ABclonal technology | Cat#A8049 |
| H3K27me3 | Cell signaling technology | Cat#9733 |
| H3K4me3 | Cell signaling technology | Cat#9751 |
| H3K9me2 | Cell signaling technology | Cat#4658 |
| H3K36me2 | Cell signaling technology | Cat#2901 |
| H3 | Cell signaling technology | Cat#4620 |

Supplementary Table 3

The sequences of si-RNA for silencing targeting genes.

| Gene Name | Sense (5'-3) | Antisense (5'-3) |
| --- | --- | --- |
| EZH2 | GACUCUGAAUGCAGUUGCUTT | AGCAACUGCAUUCAGAGUCTT |
| EZH2 | CCUGACCUCUGUCUUACUUTT | AAGUAAGACAGAGGUCAGGTT |
| SQLE | GGUGUUGUGUUACAGUUAUTT | AUAACUGUAACACAACACCTT |
| SQLE | GCAAAUCAUGCUGAACUUATT | UAAGUUCAGCAUGAUUUGCTT |
| SREBP2 | GCUGGUAAAUGGUGUGAUUTT | AAUCACACCAUUUACCAGCTT |
| SREBP2 | CCAGUGCUCUGGAGUACUUTT | AAGUACUCCAGAGCACUGGTT |
| FDFT1 | GCAGUUUCGCAGCUGUUAUTT | AUAACAGCUGCGAAACUGCTT |
| FDFT1 | GCAGUGCCUGAAUGAACUUTT | AAGUUCAUUCAGGCACUGCTT |
| LSS | GGCUGGACACCAGAAUUATT | UAAUUCUUGGUGUCCAGCCTT |
| DHCR7 | GGAAGUGGUUUGACUUCAATT | UUGAAGUCAAACCACUUCCTT |
| CYP51A1 | CGUUACAAACGAAGAUCAATT | UUGAUCUUCGUUUGUAACGTT |

Supplementary Table 4

The sequences of primers for ChIP-PCR.

| Genes | Forward | Reverse |
| --- | --- | --- |
| SREBF2-1 | TGAGGTGCTTGAAGGAGTGG | CAAGGTTGCAGATTGTCCC |
| SREBF2-2 | CGGGTGCAAAGCAGAAGACG | CCTGAGAAAGCCAGCGGAGT |
| SREBF2-3 | GTGTGACTCCGATGGCTTTC | GCCGTCCGGTCATCATCTTA |
| SREBF2-4 | TGGCGCAGATCCAACGGTGAT | GCCAATGGGCGAGCGAAGC |
| SREBF2-5 | TACTCCAGGCATTCGCTCCG | GTTTGTTGTCAATGGGACCAGG |
| SQLE-1 | GTCCTGGACACCGTGTAACC | CAAGACCAGAAACGTTGGGG |
| SQLE-2 | CCCCCTTCTCAGGACACCTA | TGTATGTGGAGAGGGCGGAA |
| SQLE-3 | GGCGCATCCGTGTATCTCTA | TCGCTTTGAGGAGAAGCCTG |
| DHCR7-1 | CCTAGGAATCTGCGTGCTCA | GATCTGGTTGCCACGTCTCT |
| DJCR7-2 | CAGAGACGTGGCAACCAGAT | GTCTGGGTGGCCTGCTTATT |
| DHCR7-3 | CCCTAATAAGCAGGCCACCC | ACGCACATTGATGGAGCGTA |
| DHCR7-4 | GTCTGTTTTGCTCCTTGCGG | ATCACCTGGATCATCGTGCC |
| DHCR7-5 | CTGAGGCACGATGATCCAGG | CCCGTGATTGGTCGAGGAG |
| DHCR7-6 | CTCAGGCTCCTCGACCAATC | GGATGATGTCAGCGATTGCC |
| HMGCR-1 | TGGGCTAAAGCCTGTTTTGGT | TGCCGCTAGAAGTCTCAAGG |
| HMGCR-2 | GGTCCTTGAGACTTCTAGCGG | GACAGACATAGTGCCTGCCTT |
| HMGCR-3 | ATGCCAACCACAGTGTGTACT | GTTCCCCACTGCTTCAGTGTA |
| HMGCR-4 | GGGAACAGTAGAACCAGCCT | CACATCATCACTCCCGACCT |
| HMGCR-5 | GGTCGGGAGTGATGATGTGT | ACCACTAATATGGCCCCACC |

Supplementary Table 5

QC of ChIP-seq

| Samples | length | Reads | Bases | Q20(%) | Q30(%) | GC(%) | N(ppm) |
| --- | --- | --- | --- | --- | --- | --- | --- |
| EZ-Input | 150.00 | 51092214 | 7663832100 | 97.46 | 93.22 | 43.96 | 10.20 |
| EZ-K27 | 150.00 | 33220928 | 4983139200 | 97.77 | 94.12 | 46.42 | 4.57 |
| NC-Input | 150.00 | 51529368 | 7729405200 | 97.48 | 93.19 | 43.62 | 10.53 |
| NC-K27 | 150.00 | 31113364 | 4667004600 | 97.86 | 94.28 | 45.59 | 4.69 |

Supplementary Table 6

QC of RNA-seq

| Samples | Total Reads | Total Tags | Total Assigned Tags | CDS Tags/Kb | Introns Tags/Kb |
| --- | --- | --- | --- | --- | --- |
| EPZ1 | 45757831 | 72524962 | 68602424 | 730.88 | 2.49 |
| EPZ2 | 49601011 | 78965945 | 74484749 | 797.38 | 2.46 |
| EPZ3 | 46841971 | 74564546 | 70447493 | 754.26 | 2.31 |
| Ve1 | 42433514 | 68037911 | 64580474 | 691.68 | 2.11 |
| Ve2 | 45345144 | 72641460 | 68704791 | 738.48 | 2.08 |
| Ve3 | 47931738 | 76797232 | 72921124 | 780.88 | 2.39 |
